# Accuracy and precision of a sphero‐cylindrical over‐refraction app for smartphones

**DOI:** 10.1111/opo.13560

**Published:** 2025-08-01

**Authors:** Rosa Maria Salmeron‐Campillo, Gines Martinez‐Ros, Jose Angel Diaz‐Guirado, Carmen Travel‐Alarcon, Mateusz Jaskulski, Norberto Lopez‐Gil

**Affiliations:** ^1^ Grupo de Ciencias de La Visión (CiViUM), Facultad de Óptica y Optometría University of Murcia Murcia Spain; ^2^ Visionapp Solutions S.L. Murcia Spain

**Keywords:** mobile application, over‐refraction, refraction, smartphone

## Abstract

**Purpose:**

To evaluate the accuracy and precision of a web‐based smartphone app designed to measure sphero‐cylindrical over‐refraction.

**Methods:**

A total of 307 healthy young subjects (22.5 ± 3.4 years) underwent clinical subjective refraction. In addition to each subject's refractive correction, spherical and cylindrical errors from −1.50 to 0.00 D were randomly induced so that their sum did not exceed −2.50 D, thereby generating a clinical over‐refraction. Seven different IOS and Android‐based smartphones were used. A new app showing tripole rescaling stimuli in blue or red light was used. Each subject used one smartphone to measure their induced over‐refraction subjectively, obtaining an app over‐refraction finding. Differences between the app and clinical over‐refraction were analysed across 675 values.

**Results:**

The mean difference between the app and clinical over‐refraction in terms of power vector components ± limits of agreement was 0.17 ± 0.84 D for M (*p* < 0.0001), 0.01 ± 0.42 D (*p* = 0.28) for J_0_ and −0.01 ± 0.32 D (*p* = 0.33) for J_45_. The corresponding values for sphere and cylinder were 0.16 ± 0.80 D (*p* < 0.0001) and 0.01 ± 0.85 D (*p* = 0.40), respectively. The sphere and cylinder measurements obtained by the app differed from the expected values by no more than 0.50 D in 85% of cases.

**Conclusions:**

The accuracy and precision of the over‐refraction obtained by the app was slightly lower than standard clinical subjective refraction in terms of inter‐examiner repeatability and comparable to measurements obtained using objective methods. The app shows the potential to perform sphero‐cylindrical over‐refractions reliably.

**Key points**

- A smartphone application‐based method for monitoring changes in the spherical and cylindrical components of refraction was evaluated.
- The mobile device measures the distance between the face and the screen during visual tasks to estimate spherical and cylindrical refraction in dioptres.
- The accuracy of the mobile application was close to standard clinical methods, with most results differing by less than 0.5 D from standard subjective refraction.

## INTRODUCTION

Uncorrected refractive errors of the eye are a great concern for public health.^1^, ^2^ Among them, myopia has been globally recognised as an emerging epidemic due to a high and increasing prevalence.^3^, ^4^ Myopia is caused by excessive eye growth (axial elongation) from childhood to early adulthood. In this period of life, it manifests as a decline in visual acuity (VA) which can be addressed by corrective lenses. Later in life, the myopic axial elongation of the eye poses an increased risk of pathology including retinal detachment.^5^, ^6^, ^7^ Therefore, early detection and monitoring of myopia are crucial to mitigate potential long‐term, irreversible consequences.

Over‐refraction refers to the lens power required to correct any remaining refractive error when a subject is wearing prescription lenses. It provides an indication of whether the lenses worn are optimal or require adjustment. In the context of myopia control in children, over‐refraction is particularly relevant since undetected residual myopia can contribute to axial elongation. Prior studies have evaluated over‐refraction in myopic children, reporting average residual spherical errors of −0.59 D with a standard deviation of 0.25 D, reaching −1.25 D in some cases, with a mean associated VA reduction of 0.24 logMAR and up to 0.50 logMAR in more extreme cases.^8^, ^9^ Considering that axial elongation in children can progress by as much as 0.6 mm (approximately −1.6 D) annually,^10^, ^11^ timely identification of any changes in refractive error may allow prompt initiation or adjustment of treatment strategies aimed at slowing myopia progression.^12^

A major contemporary challenge in optometry is simple and reliable monitoring of refraction over time. Although the conventional method of clinical subjective refraction remains the gold standard, its accessibility is limited by geographic and economic factors.^13^ At the same time, tele‐optometry allows electronic devices, such as smartphones and tablets, to conduct remote vision assessments, allowing greater accessibility to optometric services. This approach has become especially relevant in the wake of the COVID‐19 pandemic, which revealed the potential of remote healthcare solutions.^14^, ^15^ With mobile device adoption exceeding 70% globally in 2023,^16^ their high‐resolution screens, fast‐processing capabilities and built‐in sensors enable their application in vision‐related tests.^17^, ^18^, ^19^

Some mobile applications have been developed to measure refraction objectively using the rear camera and flash from a mobile device (GoCheck Kids Photoscreening; Gobiquity Inc., gocheckkids.com)^20^, ^21^, ^22^ following the principle of photorefraction,^23^ which computes the refractive error using the size of a light patch reflected at the retina.^24^ Other methodologies based on the principles of subjective refraction employ a series of screens (e.g., a smartphone screen in conjunction with a tablet or computer screen) and include Easee (Easee B.V., easee.online/en/), MyRx Refraction Exam (Luna Solutions, LLC, luna.io), Opternative (Visibly, Inc., visit.govisibly.com/opternative/), OKKO Health (OKKO Health, Inc., okkohealth.com/en‐gb/), Virtual Vision Test (Warby Parker, Inc., warbyparker.com) and an app previously evaluated by Luo et al. (EyeNexo LLC, eyenexo.com).^25^

Several challenges limit the applicability and reach of the abovementioned mobile refraction programmes. These include a lack of rigorous, peer‐reviewed clinical validation, compatibility with an ever‐changing landscape of mobile device models and their characteristics or limitations of methodology implemented in rigid user interfaces compared to the flexibility of a trained clinician.

An important challenge to overcome for any mobile device‐based refraction system is for the user to be able to perform the examination unaided and to obtain accurate results for a wide range of ametropias. Most mobile‐based instruments that perform subjective refraction estimate refractive errors by changing the distance between the eye and the device, unlike clinical methods which generally use lenses. Challenges include difficulty measuring visual acuity without correction, changes in the stimulus' spatial frequency content with varying face‐device distance, issues with hyperopic eyes (including accommodative control), screen resolution limits, assuring that the stimulus can be placed at or beyond the user's far point (FP) and the need for an interface that effectively replaces a trained clinician.

Moreover, agreement and repeatability studies are essential for validating mobile‐based refraction methods, ensuring they provide accurate results comparable to standard methods and consistent performance across repeated tests. This validation is crucial for clinical reliability, regulatory approval and professional trust. It also supports the safe integration of these technologies into routine practice, particularly in settings with limited access to traditional eye care.

The purpose of the present study is to validate a smartphone‐based subjective refraction app that operates without additional accessories and is designed to address all of the aforementioned challenges.^26^ This is accomplished by inducing different levels of ametropia (both spherical and cylindrical) in a large group of subjects without any prior experience with the app (‘naïve’ subjects) and comparing the results with those obtained by trained clinicians.

## METHODS

### Mobile devices

Seven different IOS and Android‐based smartphones were used. High screen pixel densities are required for rendering high spatial frequency (SF) visual stimuli on screen. In the present study, the minimum resolution was 391 dpi (Table 1) which allowed it to render 36 cycles per degree (cpd) (−0.08 logMAR) at a face‐device distance of 26.8 cm.

**TABLE 1:** Mobile devices used by subjects participating in the study.

Mobile device	Operating system	Screen pixel density (dpi)	Screen pixel width (μm)
iPhone 12 mini	IOS	476	53.36
iPhone 14 Pro	IOS	460	55.22
iPhone 13	IOS	460	55.22
iPhone 11 Pro	IOS	458	55.46
Samsung Galaxy S20 FE	Android	407	62.41
Vivo X60 Pro	Android	398	63.82
OnePlus Nord CE 3 Lite	Android	391	64.96

### On‐screen visual stimuli

**FIGURE 1:**
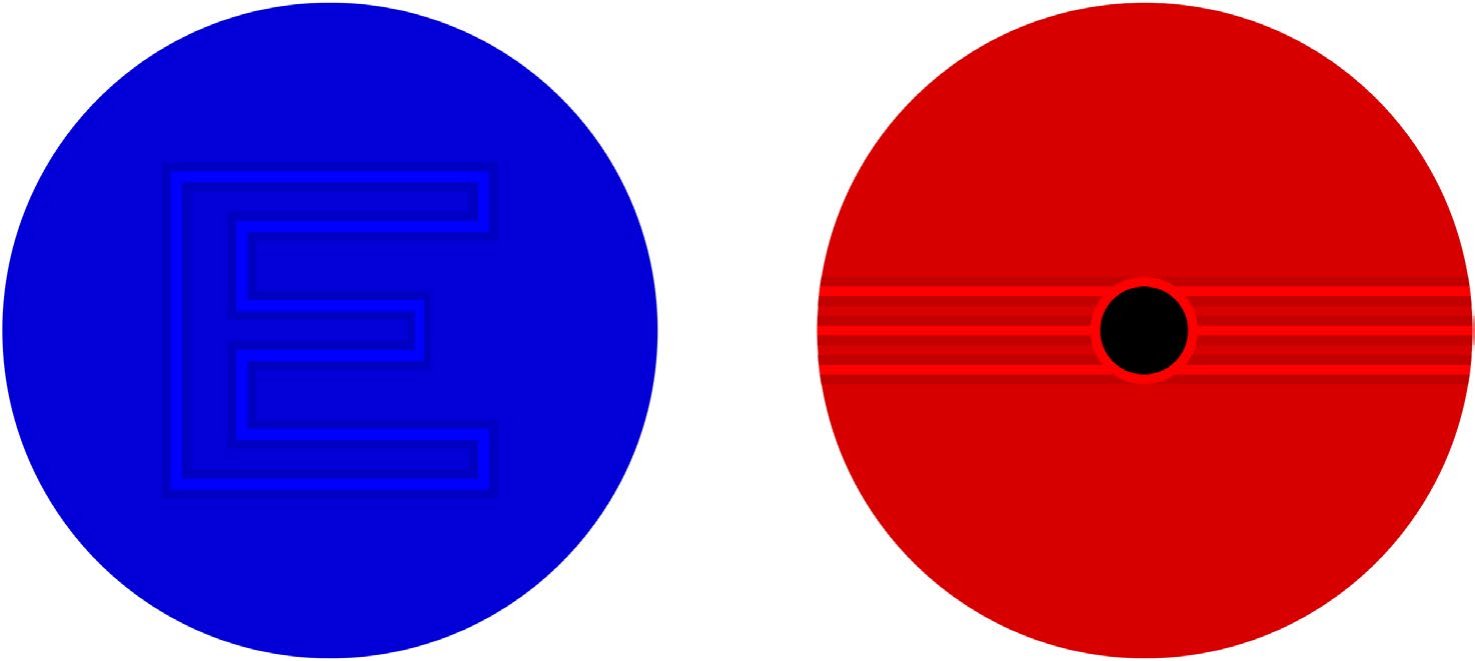
Example of the vanishing optotypes based on a tripole stimulus used by the app. Blue stimulus of a letter with a tripole contour (left) and a red stimulus with three horizontal tripoles (right).

Targets (Figure 1) consisted of letters or a set of lines which could be rotated to account for the axis of astigmatism. In either case, the stimuli were rendered using three lines (e.g., dark, bright, dark) on an intermediate coloured background. Because there are three lines and three colours, these stimuli are termed ‘tripole’ stimuli.^27^, ^28^, ^29^ It is important to note that for the line target, a central black circle was included within the lines to help the patient maintain accommodative focus, as a higher degree of precision was required during this specific measurement phase. Conversely, for the letter target, the central black dot was omitted to avoid potentially obscuring the letter's legibility, as this phase of the test aimed for a more approximate measurement.

Retinal blur depends on pupil size and can be caused by spherical and astigmatic refractive errors as well as the accommodative response. When the users increased the distance between their face and the device so that it lay beyond their FP, then a blurred image would be created at the retina, causing the target to ‘vanish’ due to insufficient retinal contrast.

### Subjects

The study included 307 young (22.5 ± 3.4 years old), healthy subjects with a wide range of refractive errors (Table 2) who were recruited between July 2024 and October 2024 from students at the University of Murcia, Spain. This sample size exceeded the minimum requirements for statistical power. All measurements were taken at the Clínica Universitaria de Visión Integral at the University of Murcia. The study was conducted with the approval of the Ethics Committee at the University of Murcia in adherence to the principles outlined in the Declaration of Helsinki.

**TABLE 2:** Age and clinical subjective refraction of subjects participating in the study.

	Age (years)	Sph (D)	Cyl (D)	M (D)	J_0_ (D)	J_45_ (D)
Mean (SD)	22.5 (3.4)	−1.2 (2.0)	−0.62 (0.57)	−1.5 (2.1)	0.13 (0.34)	0.03 (0.21)
Minimum	18	−9.50	−3.75	−9.75	−0.50	−0.70
Maximum	43	5.00	0.00	4.25	1.72	1.08

Each participant underwent both a clinical subjective and app over‐refraction. The distance correction for each participant was determined using standard clinical monocular subjective refraction guided by objective refraction (retinoscopy). The aim was to determine the maximum positive lens that provided the maximum VA (MPMVA).^9^, ^30^ VA was determined using a Bailey Lovie chart displayed at a distance of 6 m on a calibrated display (Hanion L‐1933, Hanion, manualslib.com/brand/hanion/). Peak display luminance was 230 cd/m^2^ and the Michelson contrast of the VA chart was 0.96 logMAR. The ambient illuminance was measured at the plane of the subjects' eyes using a luxmeter (HI 97500 Luxmeter, Hanna Instrument, hannainst.es) and kept consistent between clinical and app‐based measurements at approximately 250 lux. Measurements were performed monocularly with either the left or right eye, selected at random.

Subjects wore trial frames which included the optical correction using trial lenses for the measured eye and an occluder for the fellow eye. On top of this correction, refractive errors were induced using additional trial lenses. We intended to induce three refractive errors per subject, but in some cases, time constraints allowed only two measurements to be completed within the scheduled session. The induced errors were created by selecting spherical (Sph) and cylindrical (Cyl) lenses between plano and +1.50 D in such a way that the sum of the Sph and Cyl was < +2.50 D, and therefore the over‐refraction should fall in the range of plano to −2.50 D. Further, the value of the spherical equivalent (M) over‐refraction lay between plano and −2.00 D. Additionally, these cylindrical lenses were inserted with their axes at the same orientation as the subjective cylinder axis.

After a 5‐min adaptation period to the induced lenses, subjects were handed a mobile device selected at random from those listed in Table 1. Subjects followed the in‐app instructions (provided both as on‐screen text and text‐to‐speech audio) to perform app refractions. Given that each subject had two or three different induced refractive errors, a total of 675 app measurements were collected. The over‐refractions measured by the app were compared with corresponding clinical subjective refractions (clinical over‐refractions) to determine the accuracy and precision of the app.

### Determining the refractive error using the app

The app calculated the refractive error by determining the location of the FP. If astigmatism was present, then the FP was denoted as the distal (dFP) and proximal (pFP) values.

Subjective sphero‐cylindrical refraction, based upon locating the FP, has some challenges which are overcome by the *VisionApp Refraction v5* (Visionapp Solution S.L., vision.app) using a number of strategies. The first challenge is the impact of astigmatism on the spatial frequency measurements. As will be shown later, this methodology relies on measuring the maximum detectable spatial frequency (MDSF) by the user, and if astigmatism affects this measurement, then it can impact the accuracy of the results. This problem was addressed by determining initially the axis at a distance beyond the spherical equivalent FP. Following this, a set of line stimuli was presented, oriented according to both the app identified axis and the orientation perpendicular to this value. Two FP measurements are then obtained for each astigmatic meridian, that is, dFP and pFP, resulting in values for the Sph and (Sph + Cyl) meridian.

The second challenge, maintaining a constant stimulus spatial frequency, was addressed by dynamically rescaling the object after measuring the face‐to‐device distance. This approach ensures that the stimulus and its contour subtend the same spatial frequency across distances, thereby avoiding a vanishing distance of the stimulus closer to the user than its real FP in the case that the stimulus does not rescale within the distance. To this end, the images of the face captured by the smartphone camera (at 15 frames per second) are processed to obtain distances between facial landmarks (e.g., between the chin and forehead) and then converted into face‐device distances by triangulation.^18^, ^31^, ^32^

The third challenge concerns the measurement of hyperopia due to the presence of virtual FPs. This is an issue for emmetropia as well, since no real movement away from the screen would cause the vanishing optotype to disappear. This problem is addressed by leveraging longitudinal chromatic aberration (LCA) to vary the ametropia based on the colour of the stimuli (Figure 1). LCA shifts the far point closer for blue stimuli or farther for red stimuli (Figure 1). The chromatic‐influenced FP distance measurements can then be adjusted to conventional clinical standards. Specifically, the blue and red light emitted by smartphone organic light emitting diode (OLED) screens exhibits LCA of −0.67 and +0.22 D, respectively,^17^, ^33^ which results in an emmetropic eye having a FP at 1.5 m instead of infinity when viewing blue light. Moreover, a +0.5 D hyperope will exhibit a FP at 6 m in blue light, thereby allowing their refractive error to be measured. However, to avoid long face‐device distances and taking into account the potential depth of focus, in practice this method can only detect small amounts of hyperopia, typically not exceeding 0.34 D.

The fourth challenge, related to rendering high spatial frequency (SF) stimuli in smartphones, was solved by using screens with high resolution. Accurate rendering of high SFs is essential to ensure that the stimuli used for assessing VA are not limited by screen resolution, but rather by the participant's visual capabilities. When a stimulus is close to the maximum spatial frequency perceived by the subject, a more precise determination of the subject's FP will be achieved. In the present study, the minimum resolution was 391 dots per inch (dpi) (see Table 1) which allowed rendering of figures with a SF of 36 cpd (−0.08 logMAR) for a face‐device distance of 26.8 cm, which is shorter than the closest FP tested in the present study.

The final challenge, namely the absence of feedback from an eye care professional, is addressed by using a vanishing optotype test (Figure 1). This approach simplifies the process for individuals without prior experience, as they do not need to read optotypes aloud but simply move away from the phone until the stimulus disappears. Subjects indicated this endpoint (the FP) by speaking into the device, which is detected by voice recognition. Additionally, clear instructions and training procedures allowed users to complete the test without external assistance.

**FIGURE 2:**
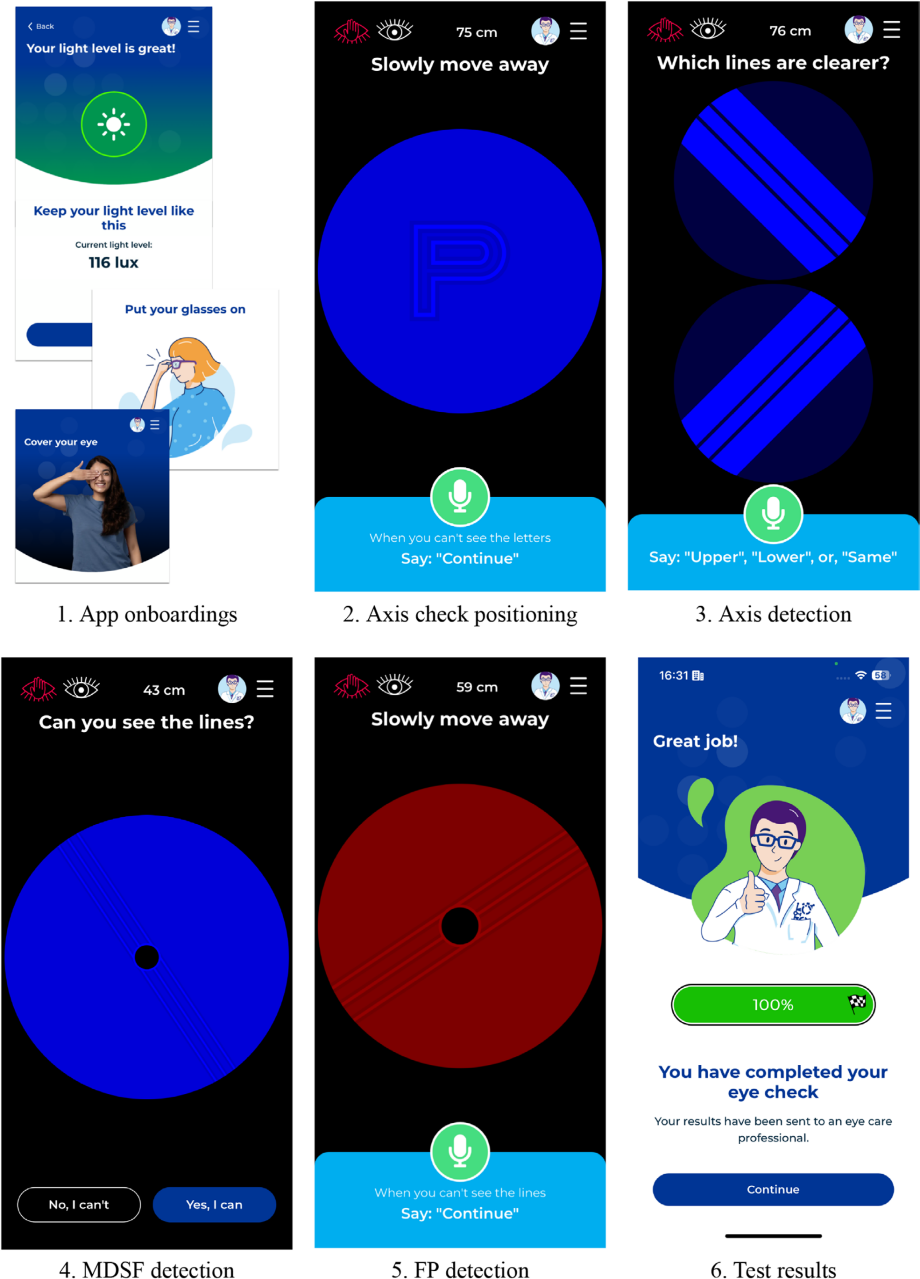
Screenshot examples of the six main stages of the app's task flow. FP, far point; MDSF, maximum detectable spatial frequency.

The general task flow for app testing was as follows (Figure 2):

1. App onboardings: The ambient light is measured to ensure appropriate conditions for testing.^34^ The face‐to‐device distance measurement functionality is set up.^18^, ^31^, ^32^ The user is instructed to use their correction, if any, and to cover one eye.
2. Axis check positioning: The subject moves away from the device beyond their interval of clear vision using a letter optotype stimulus (see left image in Figure 1).
3. Axis detection: The axis is determined using an iterative algorithm of blur comparisons for different stimuli orientations, that is, high contrast blue lines on a dark background. As with conventional clock dial tests for astigmatism, the goal is to identify the orientation of the lines that appear sharpest to the observer. However, in this digital test, this is achieved by sequentially presenting two stimuli, asking the user to compare their sharpness and identify the clearer of the two (Figure 2).
4. MDSF detection: A two‐alternative forced‐choice (2AFC) detection method is used to identify the maximum detectable spatial frequency for a group of line targets, oriented both according to the detected axis and perpendicular to that direction.
5. FP detection: Based upon the values obtained in the previous steps, the FP for each astigmatic meridian (dFP and pFP) was determined using two ‘moving‐away’ tasks involving vanishing lines. Participants were instructed to move away from the device until the vanishing lines disappeared from view. The smartphone was securely mounted on a tripod to ensure stability, and participants moved in small, controlled steps to ensure gradual separation. The endpoint was defined as the point at which participants reported complete disappearance of the lines; they were then instructed to stop and verbally confirm the disappearance before proceeding to the next measurement. The stimulus colour was initially set to red, which is appropriate for myopic subjects due to its positive LCA, causing the FP to appear farther away. However, in emmetropic, hyperopic or mildly myopic subjects (Sph > −0.75 D), this can result in an excessively distant FP. In such cases, an additional FP measurement was performed using a blue stimulus, which is more suitable for this group.
6. Computation of the app sphero‐cylindrical over‐refraction. The measured dFP and pFP distances were converted into standard clinical Sph and (Sph + Cyl) values, taking account of the LCA of the stimulus together with the axis value to obtain the sphero‐cylindrical over‐refraction.

### Statistical analysis

A sample size calculation was performed prior to data collection, using Bland–Altman analysis as the primary basis for comparison. The statistical software MedCalc (MedCalc Software Ltd., medcalc.es) was used for this calculation. The expected mean (0.00 D) and standard deviation (0.38 D) of the differences were based on the study of Carpena‐Torres et al.^35^ for values of the spherical equivalent (M). The maximum allowable difference between methods was set at 1.00 D, ensuring that it exceeded the 1.96 × standard deviation (1.96 × SD) value by 0.25 D. With α = 0.05 and β = 0.20, the required sample size was determined to be 73 participants, to provide 80% statistical power.

A Bland–Altman analysis^36^ was performed for each of the power vector (M, J_0_ and J_45_)^37^ and sphero‐cylindrical (Sph and Cyl) components, comparing the app and clinical over‐refractions. Mean difference values (related to accuracy) were calculated with their corresponding *p*‐values (with the null hypothesis being a mean difference of zero). Limits of agreement (LoA, 95%, related to precision) were calculated together with the confidence intervals (CI) for the LoA bounds.^38^ The statistical power of this analysis, determined by the number of measurements (675), resulted in a value >99%.

The differences between the measured and clinical power vector components were represented in a histogram of relative distribution and relative cumulative distribution. These differences were also computed for the cylinder axis (Axis) and its histograms were divided depending on the associated Cyl value. Additionally, the accuracy of the measured sphero‐cylindrical refractions was evaluated using a single metric, which accounts for errors in all three optometric components. This metric, which we will denote as ΔRx (also known as blur strength)^39^, ^40^ is the length of the power vector difference between the clinical and app refraction. ΔRx is an aggregation of the errors in each power vector component and has vergence units (D). The relationship between ΔRx and the differences between the app‐measured and induced over‐refraction is shown in Equation (1).

(1) $$\begin{array}{c} ∆\mathrm{Rx}=\sqrt{{\left(\mathrm{\Delta M}\right)}^{2}+{\left(\Delta J_{0}\right)}^{2}+{\left(\Delta J_{45}\right)}^{2}}=\sqrt{{\left(\text{ΔSph}+\frac{\text{ΔCyl}}{2}\right)}^{2}+\frac{1}{4}\left({\mathrm{Cyl}}_{\mathrm{App}}^{2}{\mathrm{Cyl}}_{\text{Clin}}^{2}+2\times \mathrm{Cy}l_{\mathrm{App}}\times \mathrm{Cy}l_{\text{Clin}}\cos\left(2\times \mathrm{\Delta Ax}\right)\right)} \end{array}$$

where $\mathrm{\Delta X}=X_{\mathrm{App}}-X_{\text{Clin}}$ (with *X* = {for *M*, *J*_0_, *J*_45_, *Sph*, *Cyl*, *Ax*}) is the difference between the app‐measured and the induced over‐refraction component. Histograms of the relative distribution and relative cumulative distribution for ΔRx were also computed.

Data analysis was performed using statistical computing software R 4.4.2 (r‐project.org).

## RESULTS

**FIGURE 3:**
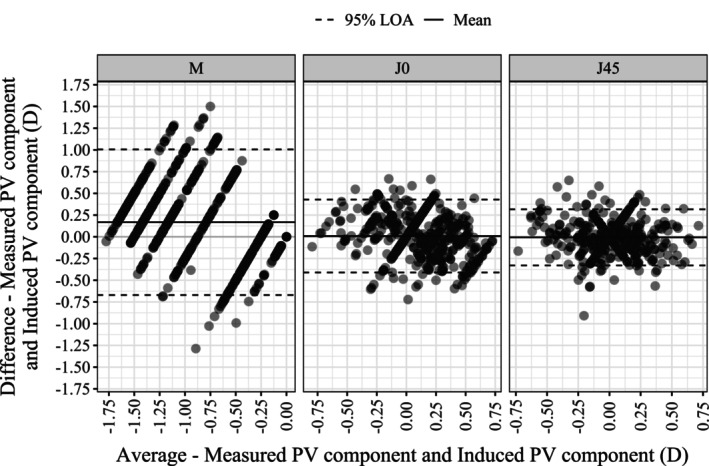
Bland–Altman analysis results for the power vector (PV) components M, J_0_ and J_45_ showing the difference between the measured and calculated over‐refraction values obtained using the app. The mean difference (bias, solid lines) and 95% limits of agreement (LoA, dashed lines) are indicated.

**FIGURE 4:**
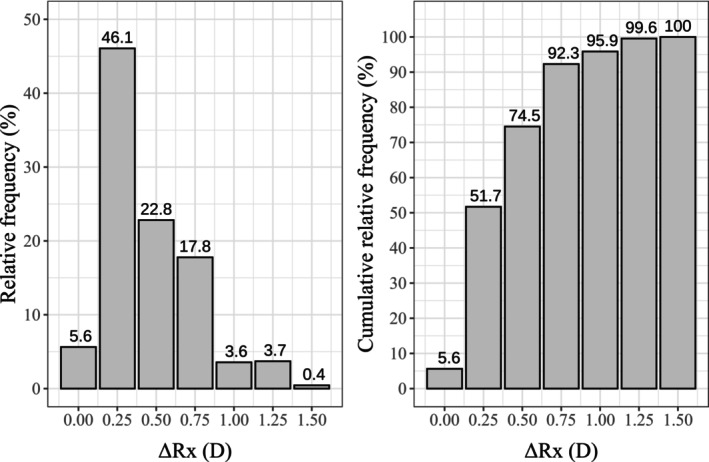
Histogram showing the relative frequency distribution (left) and cumulative distribution (right) of the blur strength metric (ΔRx) which accounts for errors in all three optometric components.

**FIGURE 5:**
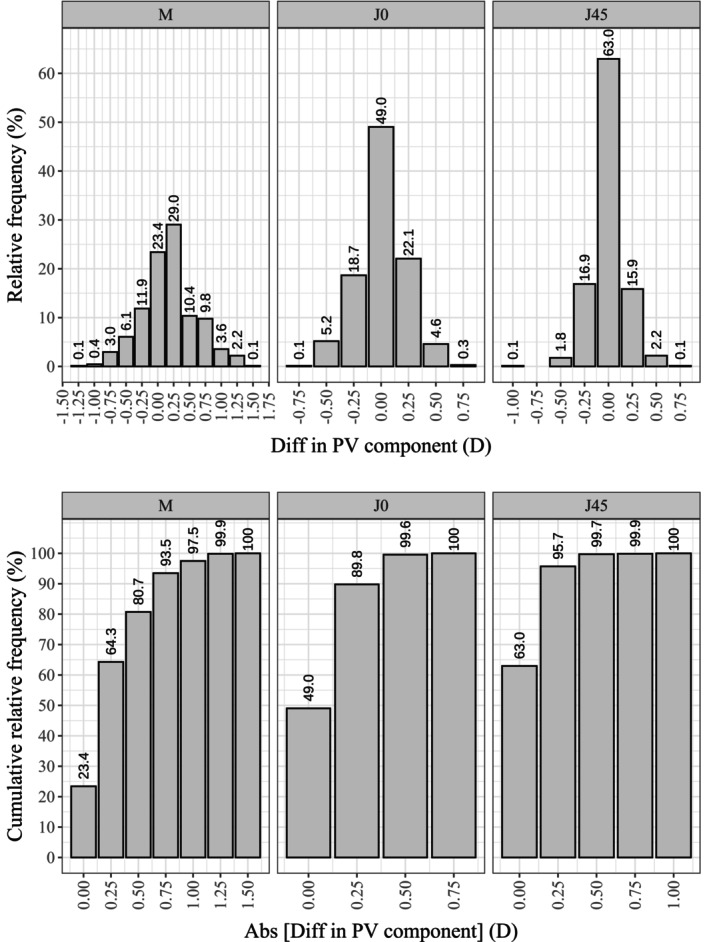
Histogram showing the relative frequency distribution (top) and cumulative distribution (bottom) of the differences in each power vector (PV) component, that is, M, J_0_ and J_45_, between the app and the standard clinical method.

**FIGURE 6:**
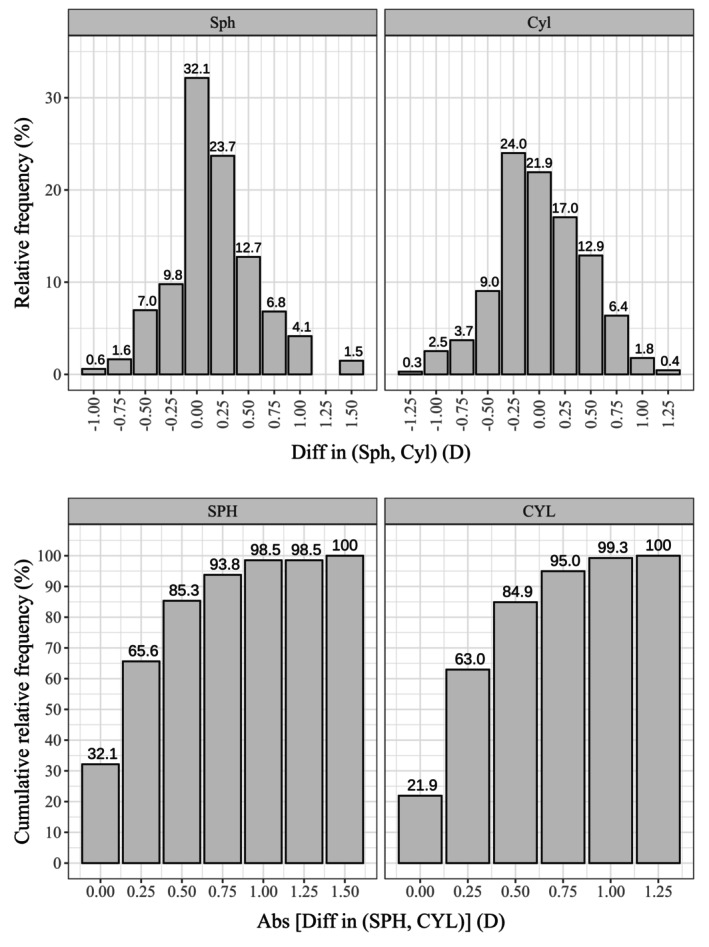
Histogram showing the relative frequency distribution (top) and cumulative distribution (bottom) of the differences in the sphere (Sph) and cylinder (Cyl) findings between the app and the standard clinical method.

**FIGURE 7:**
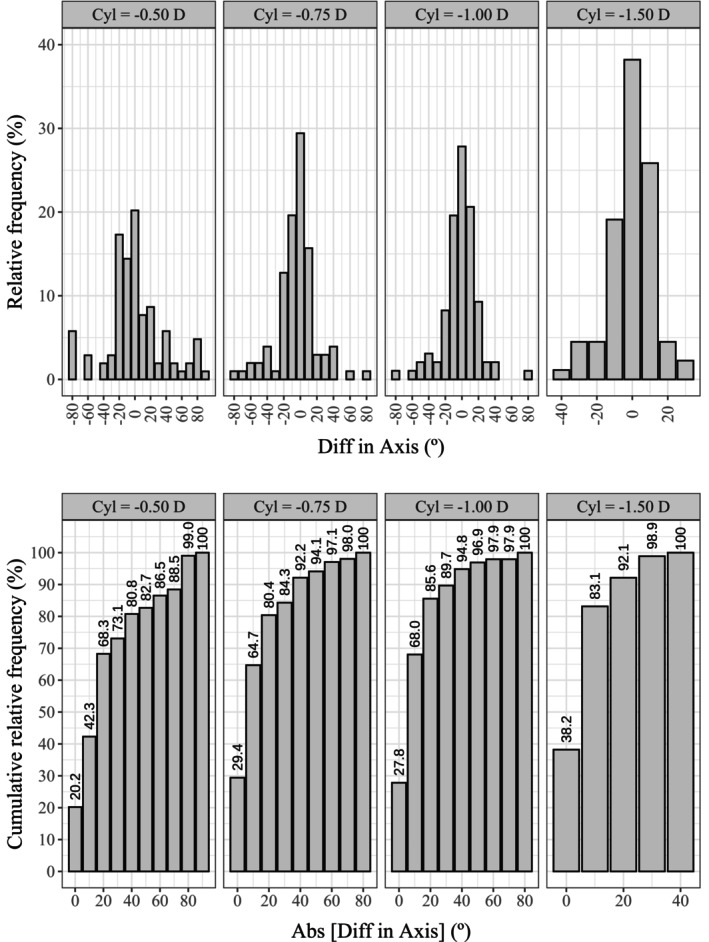
Histogram showing the relative frequency distribution (top) and cumulative distribution (bottom) of the differences in axis findings between the app and the standard clinical method, grouped by the magnitude of the astigmatism.

Figure 3 shows the results of the Bland–Altman analysis for the power vector components. The main results are also summarised in Table 3, along with the Bland–Altman analysis for the sphero‐cylindrical components. Figure 4 shows the histograms of relative frequency and relative cumulative distribution of ΔRx. Figure 5 expands on this by separating the differences for each power vector component. Figure 6 details these differences further, comparing the Sph and Cyl values between the app and clinical measurements. Finally, Figure 7 illustrates the differences in Axis, grouped by the magnitude of the Cyl.

**TABLE 3:** Summary of Bland–Altman analysis for power vectors and sphero‐cylindrical components.

	Diff mean (1.96 × SD) (D)	95% LoA (D)	95% CI of LoA (D)	*p*‐value
M	0.17 ± 0.84	(−0.67, 1.01)	LoA ± 0.055	<0.0001
J_0_	0.01 ± 0.42	(−0.41, 0.43)	LoA ± 0.028	0.28
J_45_	−0.01 ± 0.32	(−0.33, 0.33)	LoA ± 0.021	0.33
Sph	0.16 ± 0.80	(−0.64, 0.96)	LoA ± 0.053	<0.0001
Cyl	0.01 ± 0.85	(−0.84, 0.86)	LoA ± 0.056	0.40

Abbreviations: CI, confidence intervals; LoA, limits of agreement.

Note that on the relative frequency histograms, positive and negative values indicate underestimation and overestimation of the absolute value of each component, respectively.

## DISCUSSION

Generally, the results showed a good level of agreement between the app findings and standard clinical subjective refractions. In terms of the spherical equivalent (M), the app results underestimated myopia by an average of 0.17 D (*p* < 0.0001). Dispersion values, when assessed as ±1.96 × SD, were ±0.83 D. The 95% LoA (which evaluates precision) were (−0.67, 1.00) D. These outcomes can be compared with studies where the clinical results obtained by two or more independent examiners within the same population were compared (inter‐examiner). For instance, Zadnik et al.,^41^ reported an accuracy (bias) of −0.07 D and a dispersion of ±0.63 D. Their study involved 40 pre‐presbyopic healthy adults (age range 20–43 years) whose right eyes were measured on two different occasions by the same examiner using non‐cyclopegic subjective refraction. Bullimore et al.^42^ examined 86 subjects, aged 11–60 years, using both automated and clinician refraction, finding a bias of −0.17 D and a dispersion of ±0.73 D. The most comprehensive study comparing the findings of three independent optometrists was performed recently by Carpena‐Torres et al.,^35^ who reported a bias of +0.02, 0.00 or −0.02 D (depending on the comparison group) and a dispersion of ±0.75 D. Their cross‐sectional, randomised study enrolled 86 participants (mean age: 37.0 ± 18.0 years) divided into youth, non‐presbyopic adults and presbyopic adults, and each underwent three subjective refractions by three different optometrists on separate days. Therefore, the *VisionApp Refraction* results obtained here, in terms of M, are slightly less precise than the clinical subjective refraction, although the ranges of M were wider in the previous studies. It is worth pointing out that in all these inter‐examiner studies, retinoscopy was performed by all examiners. In contrast, the app subjective refractions did not begin with an objective assessment.

With regard to astigmatism (J_0_, J_45_), the mean differences between the app findings and the clinically induced values for J_0_ and J_45_ were 0.01 and −0.01 D, respectively, which were not statistically different from zero (*p* = 0.28 and 0.33, respectively). The dispersion of these terms for J_0_ and J_45_ were ±0.43 and ±0.33 D, respectively, which are similar to those obtained by Bullimore et al. (±0.38, ±0.31 D)^42^ and Carpena‐Torres et al. (±0.25, ±0.50 D).^35^ Therefore, in terms of astigmatic power vector components, the *VisionApp Refraction* results obtained here were as accurate and precise as the clinical subjective refractions.

**FIGURE 8:**
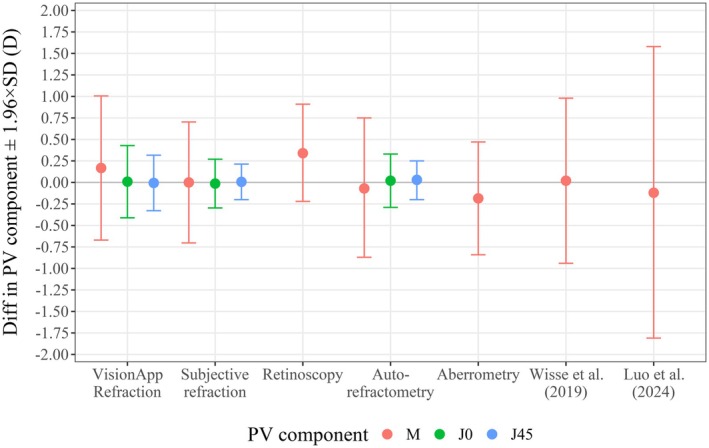
Mean (data points) and 95% limits of agreement (error bars) for the differences in the power vector (PV) components (M, J_0_, J_45_) determined using different techniques (x‐axis) and standard clinical subjective refraction. ‘Subjective refraction’ indicates the repeatability of three different optometrists performing a subjective refraction. The two right hand entries show the findings from Luo et al.^25^ and Wisse et al.^44^

Figure 8 shows a comparison between different refraction methods and clinical subjective refraction, in terms of the LoA. Objective refraction methods such as retinoscopy,^43^ auto‐refractometry^42^ and aberrometry^39^; have also been compared with the gold standard refractions. Repeatability for subjective refractions performed by different optometrists was included.^35^ Finally, the last two columns of Figure 8 represent the results of previously published studies using other refraction apps.^25^, ^44^

The precision values obtained using the different clinical refraction methods were similar. Retinoscopy showed slightly higher precision but lower accuracy.^43^ Several details of the methods compared in Figure 8 should be indicated. Subjective refraction values from Carpena‐Torres et al.^35^ indicate the mean repeatability of three different optometrists performing a subjective refraction. Aberrometry values represent the mean of 33 wavefront metrics, with mean differences from the clinical value ranging from +0.24 to −0.47 D and an average accuracy of −0.19 D.^39^ The dispersion (±1.96 × SD) of the aberrometry findings ranged between ±0.48 and ±0.93 D, with a mean dispersion of ±0.66 D (Figure 8).^39^ The range of *M* values obtained with the various techniques shown in Figure 8 varied considerably. For instance, the values of *M* in Wisse et al. ranged from −3.00 to 0.00 D,^44^ while Luo et al. tested myopic subjects up to −8.00 D.^25^ Note that although Luo et al. examined a broader range of M, this subset was included to ensure comparability with the present study.

Major challenges commonly associated with mobile‐based subjective refraction were addressed in the present approach. Astigmatism was managed by determining the axis beyond the interval of clear vision and performing meridional FP measurements. Variations in stimulus size due to differences in the face‐to‐device distance were corrected by dynamic rescaling based on real‐time facial tracking. Hyperopic and emmetropic far points, which are virtual or infinite, were rendered measurable using chromatic aberration to induce controlled ametropia. High spatial frequency rendering of the target was ensured by using smartphones with sufficient pixel density. Finally, user autonomy was enabled through a vanishing optotype task, with voice input and guided instructions, thereby reducing the need for professional supervision.

One limitation of the study is the relatively small M range tested: −2.00 D to plano. Regarding negative values of M, although −2 D may seem a small myopic value, it should be kept in mind that the primary goal of the tested app is to detect the development or progression of myopia or astigmatism, that is the sphero‐cylindrical refraction or over‐refraction. In this regard, it seems unlikely, especially in developed countries, that an individual would present with more than 2 D of uncorrected myopia,^8^ which would likely correspond with uncorrected VA worse than 0.50 logMAR.^8^ Indeed, reports of the first clinical refraction rarely show values of sphere and cylinder < −2.00 and −1.50 D, respectively.^8^ Moreover, in areas where myopia progression is rapid, such as Asia, it is uncommon to find a change greater than 1 D per year. A further limitation is that the study assessed accuracy and precision but not repeatability. Although repeatability is encompassed within precision, it was not evaluated separately. This could be explored in future research.

Regarding hyperopic refractions, it would be interesting to measure these individuals, as decreasing hyperopia could lead to the development of myopia. As mentioned in the Methods section, the use of black and white stimuli on a smartphone without additional lenses makes it impossible to obtain a direct measurement of hyperopia. However, there are two techniques that could partly overcome this limitation. The first is the use of blue stimuli, which would allow for refraction testing up to around +0.25 D with a smartphone placed at 2.38 m (0.25 D = 0.67 D –1/2.38 m, where 0.67 D is the LCA in blue light)^17^ or even +0.50 D with the smartphone placed at 6 m, if technological limitations at these distances were overcome. This would theoretically allow the measurement of very small amounts of hyperopia. The second consists of the use of the smartphone to find the near point of accommodation. This parameter is not only useful in presbyopia,^45^ but allows the user to obtain an assessment of the refraction due to the strong relation between the amplitude of accommodation and age. This is patented methodology that represents a promising new way to measure hyperopia with a smartphone, beyond the scope of the present study.^26^ However, there could be some limitations in this methodology, such as the dispersion in amplitude of accommodation for subjects of a certain age.^46^, ^47^

In conclusion, this study evaluated a novel smartphone app for measuring myopia and astigmatism subjectively. The accuracy and precision were comparable to standard clinical subjective refractions in terms of inter‐examiner repeatability, within the range of refractive errors studied. Accuracy was high, with biases less than 0.25 D for all power vector components. Precision was also satisfactory, with the app's LoA for any power vector component being no more than 0.25 D greater than those observed for clinical subjective refraction.^35^ Therefore, it may be concluded that this device has potential for monitoring the progression of myopia and astigmatism, thereby allowing early prevention and effective management.

## AUTHOR CONTRIBUTIONS

**Rosa Maria Salmeron‐Campillo:** Conceptualization (equal); investigation (lead); methodology (lead); project administration (lead); writing – original draft (lead); writing – review and editing (lead). **Gines Martinez‐Ros:** Data curation (lead); formal analysis (equal); software (equal); writing – original draft (equal). **Jose Angel Diaz‐Guirado:** Data curation (supporting); formal analysis (equal); software (supporting). **Carmen Travel‐Alarcon:** Methodology (equal); resources (supporting); software (supporting); validation (supporting); visualization (supporting). **Mateusz Jaskulski:** Conceptualization (supporting); resources (lead); software (lead); writing – review and editing (supporting). **Norberto Lopez‐Gil:** Conceptualization (lead); supervision (lead); validation (lead); visualization (lead); writing – original draft (supporting); writing – review and editing (supporting).

## FUNDING INFORMATION

This research received no funding.

## CONFLICT OF INTEREST STATEMENT

RMSC: Employee, Visionapp Solutions SL, GMR: Employee, Visionapp Solutions SL, JADG: Employee, Visionapp Solutions SL, CTA: Employee, Visionapp Solutions SL, MJ: Owner, Employee, personal financial interest in Visionapp Solutions SL, NLG: Owner, Consultant, personal financial interest in Visionapp Solutions SL.

## Data Availability

Data underlying the results presented in this paper are not publicly available at this time but may be obtained from the authors upon reasonable request.
